# The Profession of Medicine

**Published:** 1852-05

**Authors:** 


*The Profession of Medicine.*—We copy the following letter from Prof. Lee as an act of justice to himself and the University of Buffalo with which he is connected. It was addressed to the editor of the *Ohio Medical Journal* and explains itself.

MY DEAR SIR: In the last number of your Journal I notice, in a review of Dr. Wood's, "*Hints to the People upon the Profession of Medicine*," published by Mr. Derby, of this city, some animadversions upon myself and colleagues of the Buffalo Medical College, for a recommendation of the same, as found in your advertisements. It is due to myself to say that my name was appended to said recommendation without my knowledge and consent, and while I was absent in New York. I am also authorized in saying that Professor Hadley, and probably others, whose names are appended thereto, never saw the paper until published in your Journal.

The remarks you have quoted, doubtless, do great injustice to our medical schools, and the character of medical students, and are altogether unworthy of the author of the essay in which they appear. I disapprove of them as much as you do, and regret that, through inadvertance, probably, they were allowed to go forth to the world; for I have so much confidence in the candor and good sense of the author, that I have no doubt he would have suppressed them, had he given the matter a little reflection. But still, they are but as spots on the disc of the sun, resplendent with light and glory; and as I look upon the essay as a whole, I see there specks, no more than I do the natural spots on the sun's surface, which require a smoked glass, or a telescope, in order to bring them into view. Had I been applied to, I am free to say, I should have recommended the essay; not supposing, however, that by so doing, I should have been understood as endorsing every sentiment and statement therein contained, just as you publish many things, which you would hardly be willing to endorse, and perhaps decidedly disapprove. We must be a little charitable towards each other, and not be hasty to condemn where the motives appear to be praiseworthy. It has been the fashion with some uneasy spirits to find fault with medical schools and medical education; to run down every thing indigenous, and praise every thing of exotic growth; to preach incessantly—*reform, reform,* without the slightest effort to reform themselves, or do any thing to advance the progress of medical education, or improve the state of the profession. It is the easiest thing in the world to grumble and find fault, and more individuals, doubtless, suppose that by so doing they show their own superiority; but all honorable and candid minds draw a different conclusion. If we look at the state of the medical profession in the United States, as it existed twenty years ago, and compare it with the present, we see a most astonishing advance in medical education, greater than can be shown in any other profession or calling, and the change must chiefly be attributed to the superior instruction furnished by our Medical Colleges. An improvement has been constantly going on, and if the *croakers* would but lend a hand, and be a little more particular with regard to the character, and preliminary education and qualifications of those whom they admit into their offices as private students, we should in a few years see a still more rapid improvement, and we should not be accused of graduating those who are unfit to be enrolled as members of the profession.

The Israelites would doubtless have furnished good brick, if they had only had *straw,* as our Medical Colleges will turn out good doctors, if they have the proper materials. I would as soon think of making the axle of a rail-road car out of white-wood, or a dwelling of corn-cobs, or seeing wooden nutmegs fructify, as a scientific, well-qualified practitioner out of some of the material that is sent us; and yet we are required not only to furnish instruction, but brains; to furnish the inner, and polish the outer man; to improve the morals, and finish the manners; to turn out, in short, none but Sydenhams and Borhaaves, Hunters, Gregorys, Coopers, Lawrences, Rushes and Godmans. Well, we will do all we can; but our brethren must not be too exacting; in general they are not; the grumblers are comparatively few, and yearly growing fewer; but we beseech them to be a little more particular as to the material which is supplied to our medical schools; for corn does not yield wheat flour, nor squash-seed, water-melons; nor cheat change into wheat. Let them but do their part, we will then do ours. Let us cultivate a good understanding, and pull all together, and not stand grumbling and fault-finding, when there is need for united and constant exertion. But, to return to Dr. Wood's essay,—I am willing to have it go forth as it is: though I should prefer to see the unsightly excrescences you have pointed out lopped off. It is calculated to do great good, and I desire, therefore, to see it widely circulated. A physician whose beat is infested with piratical marauders and interlopers, quacks, who are trying to climb up some other way, and poach upon their honest neighbor's possessions, should go armed with these pamphlets for gratuitous circulation. They will prove as fatal to the whole tribe as arsenic to rats; whether they be homœopathic, hydropathic, eclectic, botanic, or mesmeric. Their name is legion. I am willing to risk any contingent evil, for the certain good which they will produce. The essay shows a masterly mind—a comprehensive, philosophical, far-reaching intellect, which views a subject in all its bearings and relations, and illustrates with the clearness of a sun-beam. Honor then, to whom honor is due; and let us not seek for smoked glass, or magnifying lenses, to discover specks and spots; but hail the blessed light, and rejoice in its beams.

Truly, your friend,

CHARLES A. LEE, M. D."

*Buffalo, January* 26, 1852.

